# Emergency correction of coagulation before major surgery in two elderly patients on oral anticoagulation

**DOI:** 10.1186/1477-9560-5-1

**Published:** 2007-01-10

**Authors:** Marzia Angelo, Bernhard Gutmann, Michele Adami, Bernd Zagler, Anton Zelger, Christoph Pechlaner, Christian J Wiedermann

**Affiliations:** 1 2^nd ^Division of Internal Medicine, Department of Internal Medicine, Central Hospital of Bolzano, Bolzano, Italy; 2 Division of General Internal Medicine, Department of Internal Medicine, Medical University of Innsbruck, Innsbruck, Austria

## Abstract

Recommendations for urgent reversal of oral anticoagulation with vitamin K_1 _antagonists are largely derived from case series employing empirical dosing regimens with vitamin K_1 _and prothrombin complex concentrates. Data on the use of prothrombin complex concentrates in this indication are scarce in the elderly who are at high risk of both hemorrhagic and thrombotic complications. The two cases presented here describe patients older than 75 years who underwent rapid International Normalized Ratio (INR) reversal with prothrombin complex concentrates for surgical treatment of a bleeding ruptured spleen and for emergency surgery of a dissecting aorta. Both patients had their INRs rapidly corrected to ≤ 1.6 and underwent operation without complications. Evidence on treatment of patients who present with elevated INR and who have major bleeding or need to undergo emergency surgery is based mainly on observational studies. The two elderly patients presented here underwent successful emergency surgery after their INRs had been corrected with the intravenous use of vitamin K_1 _in combination with prothrombin complex concentrate that was administered according to current guideline recommendations.

## Background

For "over-anticoagulation" associated with major bleeding or in the case of reversal for emergency surgey in patients orally anticoagulated with coumarin or related vitamin K_1 _antagonists, administration of vitamin K_1 _is inadequate, since its full effect occurs after 12 to 24 hours. The volume of plasma required for reversal is almost invariably too large to be infused safely, whereas prothrombin-complex concentrates (PCC) are effective and convenient [1,2]. These products are virally inactivated and therefore safer than plasma with respect to the transmission of viral diseases, but a thrombogenic effect has occasionally been reported; as bleeding complications are more prevalent in orally anticoagulated elderly patients, a lack of published data on the clinical use of PCC in the elderly may even be more relevant regarding its safety [3-5]. Some randomised controlled studies on the safety of administering a PCC to urgently reverse the anticoagulant effects of vitamin K_1 _antagonists to normalize INRs support the recommendations of consensus guidelines developed by various task forces and expert panels that PCCs are the agents of choice for urgent vitamin K_1 _antagonist reversal [3].

We describe our experience with two patients older than 75 years of age in whom PCC was used for immediate correction of their INRs before undergoing major cardiothoracic and abdominal emergency surgery.

## Cases

### Patient 1

A 80 year-old patient receiving coumarin for atrial fibrillation, arterial hypertension and diabetes mellitus was admitted to our hospital because of left-sided thoraco-abdoiminal pain. Initial evaluation ruled out coronary artery disease. An electrocardiogram on admission demonstrated atrial fibrillation at a rate of 145 beats per minute. There was no hematemesis or melena. Presence of low-grade fever and non-productive cough, and the physical examination suggested lower airway infection which was confirmed by chest radiography. Blood tests revealed anemia with hemoglobin 10.4 g/dL and hematokrit 31.4%, leukocytosis of 10,600 G/L and an elevated C-reactive protein of 14.9 mg/dL. Under long-term oral anticoagulation with coumarin an actual INR of 2.96 was determined. His pain presented with slow onset and was aggravated by respiration suggesting its pleural origin due to lower respiratory tract infection. As after 48 hours pain was continuously present and now independent of respiration, additional imaging studies were performed. Abdominal ultrasonography and computerized tomography identified splenomegaly with inhomogenous parenchymal structure compatible with splenic hemorrhage. Hemodynamically stable his hemoglobin had dropped to 8.3 g/dL. Therefore PCC ("Uman Complex^®^"; Kedrion Spa., Castelvecchio Pascoli, Italy) at a dose of 50 IU per kg body weight were administered i.v. in combination with 10 mg of vitamin K_1_. At three and a half hours after PCC infusion, the INR was 1.5 and emergency splenectomy was performed. Surgical intervention and postoperative course were uncomplicated with no signs and symptoms of venous or arterial thromboembolism. The patient was discharged after oral anticoagulation had been reinstituted.

### Patient 2

A 77-year-old man without a particular family history but with a past medical history of peripheral arterial occlusive disease that was surgically treated including long-term oral anticoagulation with coumarin presented with sudden onset chest discomfort at rest. He was admitted to our hospital. Hemodynamics were stable. There were no rales or heart murmurs, except S4 of the heart sound. Electrocardiography showed sinus rhythm with non-specific ST-segment changes in leads I, II, aVL, aVF and V3–6. Arterial blood pressure was 130/100 mmHg on the right arm and 110/60 mmHg on the left. The chest pain initiated with a blow and then migrated toward the lumbar area over time. Chest X-ray was unremarkable but contrast computed tomography confirmed aortic dissection from the ascending aorta to the abdominal aorta. Stanford type A aortic dissection with a possible tear entry near the aortic arch was diagnosed. The patient was oliguric 8 hours after admission. No other symptoms suggesting cerebral, spinal or peripheral perfusion deficits or hematoma formation were present. Laboratory examination identified elevated D-dimer levels and an INR of 3.44; other blood tests including cardiac markers and renal function tests were normal. PCC at a dose of 45 IU per kg body weight were administered i.v. in combination with 10 mg of vitamin K_1_. He was then transferred to Verona University Hospital approximately 10 h after the onset. Two and a half hours after intravenous administration of PCC the INR was 1.6. Emergency graft replacement of the ascending aorta was performed. Surgical therapy was performed without complication and the postoperative clinical course was uneventful. Renal other organ functions remained normal and unaffected, respectively. The patient was transferred to his home hospital in Germany on the 6^th ^postoperative day in stable condition after oral anticoagulation with coumarin was again begun.

## Discussion

The two cases presented here describe orally anticoagulated patients over 75 years of age whose prolonged INRs were corrected with PCCs for surgical treatment of a bleeding ruptured spleen and for emergency surgery of a dissecting aorta. Both patients had their INRs corrected to below 1.5 and underwent operation without excessive bleeding or thromboembolic events.

Limitations in the use of PCC for anticoagulant reversal include the elderly because many studies reported orally anticoagulated patients mainly <75 years of age who are at lower risk of both, bleeding as well as atherothrombotic complications [5]. Current recommendations for emergency anticoagulant reversal apply to adult patients independent of age [1-4]. In the two presented cases with patients of > 75 years of age, PCC was effective and safe in reversing oral anticoagulation.

As the INR increases with oral anticoagulation, it has been assumed that any approach to the normalization of increased INRs is equivalent to reversal of the anticoagulant effect and the re-establishment of normal hemostasis. Even though the various strategies and therapeutic agents available to achieve this goal have rarely been compared in head-to-head randomized controlled clinical trials, case series including reports like this one are in support of using INR as a valid surrogate marker for corrected hemoastasis.

Thrombotic episodes have been reported with PCC when they have been used to cover surgery in patients with haemophilia B and in patients with liver disease [6]. Evans et al., however, reported zero thrombotic events in 59 patients from three studies where PCCs were used to reverse warfarin and estimated the upper 95% confidence interval for thrombosis post-PCC treatment to be 6% or 1 in 16 [7]. As always, a risk benefit decision has to be made for each patient, but, in the presence of major bleeding or emergency surgery, the benefits are likely to always exceed the small risk of thrombosis. No evidence of thromboembolic complications were seen in the two patients reported with multiple risk factors for developing such adverse events.

The optimal method for returning the INR to the desired range depends upon its degree of initial elevation and whether or not clinically significant bleeding is present. Recommendations have been elaborated by the American College of Chest Physician Consensus Conference and others [1,2] which are based on studies that use the elevated INR as a surrogate marker for the risk of bleeding. Most of the evidence has been obtained for anticoagulation with warfarin with evidence for phenprocoumon being less well established [2]. Evidence on treatment of patients who present with elevated INR and who have major bleeding are based mainly on observational studies. Treatment includes withholding oral anticoagulants, administering intravenous vitamin K, and transfusion of coagulation factor concentrates (Table 1). Coagulation factor replacement in combination with vitamin K therapy is currently recommended in patients with coumarin-associated major bleeding or with indication for immediate correction of their INR [2,5]. PCCs contain coagulant factors II, VII, IX, and X and can reverse the effect of coumarin more rapidly than fresh-frozen plasma in coumarin-related coagulopathy [10-14]. The PCC used in our patients (Uman Complex; Kedrion Spa., Castelvecchio Pascoli, Italy) contains factors II, IX and X, and reversed the INR to levels allowing emergency surgery at about 1.5. Other PCCs contain also factor VII (e.g. Beriplex P/N; ZLB Behring, Marburg) and may offer faster achievement of full anticoagulant reversal in even a smaller period of time.

**Table 1:** Management of elevated INR or bleeding in patients treated with vitamin K antagonists targeted at an INR range of 2.0 – 3.0*

3.0 < INR ≤ 3.5 (no bleeding)	No dose reduction may be required Monitor INR again before lowering the dose
3.5 < INR ≤ 5.0 (no bleeding)	Omit dose Monitor daily and resume at lower dose when INR in therapeutic range
5.0 < INR < 9.0 (no bleeding)	Omit dose Give oral vitamin K_1 _(phytomenadione) 1 – 2.5 mg. Monitor daily and resume at lower dose when INR in therapeutic range
INR ≥ 9.0 (no bleeding)	Hold vitamin K antagonist therapy Give oral vitamin K_1 _at higher dose (5 – 10 mg) with the expectation that the INR will be reduced substantially in 24 to 48 hours Monitor daily and give additional vitamin K_1 _if necessary Resume at lower dose when INR in therapeutic range Hospitalization may be considered if patient at higher risk of bleeding
Major bleeding at any elevation of INR	Hospitalization Hold vitamin K antagonist therapy and give prothrombin concentrate complex supplemented with vitamin K_1 _(10 mg by slow intravenous infusion) Monitor from the fouth hour after prothrombin complex concentrate

* Modified after Ref. [2, 14]

In conclusion, vitamin K_1 _antagonist-associated coagulopathy is commonly encountered and is particularly associated with bleeding in the elderly [4]. Avoiding over-anticoagulation and reducing periods of overdosing in the course of oral anticoagulant treatment may help to minimise the risk of bleeding. Evidence on treatment of patients who present with elevated INR and who have major bleeding are based mainly on observational studies. Randomized clinical trials that measure the usefulness of interventions by clinical mortality and morbidity (bleeding and thrombosis) endpoints are required and under way. Optimal treatment, including the use of oral vitamin K_1 _in the asymptomatic patient, and intravenous vitamin K_1 _in concert with coagulation factors in the bleeding patient, should reduce the mortality associated with this condition.
